# Limbic system damage in MS: MRI assessment and correlations with clinical testing

**DOI:** 10.1371/journal.pone.0187915

**Published:** 2017-11-09

**Authors:** Jie Wen, Dmitriy A. Yablonskiy, Amber Salter, Anne H. Cross

**Affiliations:** 1 Mallinckrodt Institute of Radiology, Washington University School of Medicine, St. Louis, MO, United States of America; 2 Division of Biostatistics, Washington University School of Medicine, St. Louis, MO, United States of America; 3 Department of Neurology, Washington University School of Medicine, St. Louis, MO, United States of America; Nathan S Kline Institute, UNITED STATES

## Abstract

Volume loss in some limbic region structures has been observed in multiple sclerosis (MS) patients. However, in vivo evaluation of existing tissue cellular microstructure integrity has received less attention. The goal of studies reported here was to quantitatively assess loss of limbic system volumes and tissue integrity, and to evaluate associations of these measures with cognitive and physical dysfunction in MS patients. Thirty-one healthy controls (HC) and 80 MS patients, including 32 relapsing remitting (RRMS), 32 secondary progressive (SPMS) and 16 primary progressive (PPMS), participated in this study. Tissue cellular integrity was evaluated by means of recently introduced tissue-specific parameter R2t* that was calculated from multi-gradient-echo MRI signals using a recently developed method that separates R2t* from BOLD (blood oxygen level dependent) contributions to GRE signal decay rate constant (R2*), and accounting for physiological fluctuations and artifacts from background gradients. Volumes in limbic system regions, normalized to skull size (NV), were measured from standard MPRAGE images. MS patients had lower R2t* and smaller normalized volumes in the hippocampus, amygdala, and several other limbic system regions, compared to HC. Alterations in R2t* of several limbic system regions correlated with clinical and neurocognitive test scores in MS patients. In contrast, smaller normalized volumes in MS were only correlated with neurocognitive test scores in the hippocampus and amygdala. This study reports the novel finding that R2t*, a measure that estimates tissue integrity, is more sensitive to tissue damage in limbic system structures than is atrophy. R2t* measurements can serve as a biomarker that is distinct from and complementary to volume measurements.

## Introduction

Cognitive impairment, including impairments of memory, attention, information processing and executive functions, affects approximately 50% of multiple sclerosis (MS) patients [1]. Dysfunctions of episodic memory and information processing speed are the most common types of dysfunctions reported [2]. The limbic system contains structures that play important roles in memory functions. These include not only the hippocampus but also the amygdala, which has roles in cognition and memory processing as well as in emotion [3,4]. Previous studies in MS observed atrophy in parts of the limbic system [5,6] which was associated with memory impairment [7]. Abnormalities in the white matter (WM) tracts connecting structures of the limbic system, such as the cingulum, fornix and uncinate fasciculus, have been demonstrated in MS using diffusion tensor imaging, and the abnormalities correlated with cognitive dysfunction [8–10].

We developed a technique called gradient echo plural contrast imaging (GEPCI), which is a post-processing technique that is based on a commercially-available gradient recalled echo (GRE) pulse sequence with multiple gradient echoes [11–13]. GEPCI can provide quantitative measurement of R2*, which is the relaxation rate constant of the transverse magnetization which measures how fast the MR signal decays. We previously analyzed R2* to evaluate global and regional GM integrity in MS, finding that lower R2* in cortical GM correlated with worse neurocognitive test scores [14]. R2* and quantitative susceptibility mapping (QSM) have been found to be sensitive to iron deposition in basal ganglia and were correlated with inhibitory control in MS patients [15]. In studies reported in this paper we used MRI-based methods to measure tissue-specific relaxation properties of GRE MRI signal, i.e. the R2* (1/T2*) relaxation rate constant and its sub-component, R2t*, specifically related to tissue cellular structure [16,17]. The R2* was demonstrated to detect WM abnormalities in MS patients, progressively decreasing with myelin loss [18]. Besides tissue-specific contributions, susceptibility related effects, such as extravascular BOLD (blood-oxygen-level dependent) effect which is continually changing in response to neuronal activity [19], also contribute to R2* signal. Our group introduced a method [16] to separate tissue-specific (R2t*) from BOLD [20] contributions to R2*. The tissue-specific (R2t*) MRI relaxation parameter depends on the cellular environment of water molecules (the main source of MRI signal). Higher concentrations of proteins, lipids, and other components of biological tissue and cellular constituents (sources of MR signal relaxation) lead to higher relaxation rate constants. For example, in pure water or CSF, the R2t* is about 1 s^-1^, while in normal brain tissue it is about 15–20 s^-1^. Using this method [16] and acquisition plus post-processing techniques that minimize artifacts related to macroscopic magnetic field inhomogeneities [21], and physiological fluctuations [22], we previously demonstrated that R2t* measurements are sensitive to age-related changes in brain tissue cellular structure in healthy adults [17] and loss of tissue cellular integrity in Alzheimer Disease [23]. By the elimination of the variable contribution to R2* that is dependent on venous blood oxygenation level (BOLD), R2t* values provide more tissue specific information than R2* [17,23].

Here we measured R2t* and volumes of limbic system components–with a focus on hippocampus and amygdala–in 80 MS patients and 31 healthy controls (HC). We observed that not only did MS patients have smaller hippocampus volumes compared to HC, but R2t* in hippocampus and amygdala and several other limbic regions was also reduced, suggesting damage even in non-atrophied tissue. Moreover, alterations in R2t* and volume of several different regions in the limbic system of MS patients correlated with clinical and neurocognitive test scores.

## Materials and methods

### Participants

The study was approved by the Institutional Review Board of Washington University School of Medicine. Eighty MS patients with relapsing remitting (RRMS, n = 32), secondary progressive (SPMS, n = 32) or primary progressive (PPMS, n = 16) clinical courses were enrolled. Ten male and 21 female HC, age 23 to 85 years (mean age ± SD: 49.53 ± 15.98), were enrolled to represent the age range of the MS patients (Table 1). Sixty out of 80 MS patients were on disease-modifying treatments, including beta-interferon (n = 20), dimethyl fumarate (12), fingolimod (4), glatiramer acetate (9), methotrexate (1), natalizumab (4), ocrelizumab (5) and teriflunomide (5). All subjects provided written consent.

**Table 1:** Participant characteristics.

		HC	RRMS	PPMS	SPMS
**Number of participants**	F	17	27	10	20
	M	9	5	6	12
**Age**	F	53.00±14.54	54.70±8.01	56.40±8.60	57.45±8.71
	M	42.23±17.17	51.80±16.27	53.50±10.43	56.67±10.79
**EDSS**	F	N/A	2.70±1.34	5.70±1.18	6.03±1.23
	M	N/A	2.00±0.71	5.75±1.04	5.29±1.48
**25FTW**	F	N/A	5.07±1.63	15.77±26.65	13.88±8.90
	M	N/A	3.87±0.93	12.62±10.66	9.35±5.77
**9HPT (Dom)**	F	N/A	22.58±7.11	28.82±19.40	28.30±16.59
	M	N/A	23.42±4.90	31.23±7.79	36.03±11.09
**9HPT (Non Dom)**	F	N/A	22.16±4.39	29.32±15.17	37.10±29.88
	M	N/A	20.96±2.84	47.21±49.91	39.97±19.60
**PASAT (3s)**	F	N/A	45.89±11.97	47.90±11.12	42.44±14.12
	M	N/A	49.40±6.80	51.00±5.73	42.00±9.81
**PASAT (2s)**	F	N/A	34.00±10.05	37.10±8.32	32.06±12.34
	M	N/A	38.60±5.37	42.67±9.63	30.33±8.69
**SDMT**	F	N/A	53.07±11.84	51.20±9.15	43.16±12.48
	M	N/A	55.00±4.53	51.17±14.34	40.75±13.85

Mean ± SD for each parameter was shown in the table. HC = Healthy Control; RRMS = Relapse Remitting Multiple Sclerosis; PPMS = Primary Progressive Multiple Sclerosis; SPMS = Secondary Progressive Multiple Sclerosis; EDSS = Expanded Disability Status Scale; 25FTW = Timed 25-Foot Walk; 9HPT (Dom) = 9-Hole Peg Test of the dominant hand; 9HPT (Non Dom) = 9-Hole Peg Test of the non-dominant hand; PASAT = Paced Auditory Serial Addition Test; SDMT = Symbol Digit Modalities Test.

### MRI

Scans were performed using a 3T Trio MRI scanner (Siemens, Erlangen, Germany) with a 32-channel phased-array head coil. High resolution GEPCI [13] datasets of voxel size 1×1×2 mm^3^ were acquired using a three dimensional multi-gradient-echo sequence with flip angle of 30°, TR = 50 ms and total acquisition time of 12 min. For each acquisition, 10 echoes were collected with first echo time TE1 = 4ms and echo spacing ΔTE = 4ms. Navigator echoes were collected to correct physiological fluctuation-induced artifacts [22]. A voxel spread function algorithm [21] was used to correct for artifacts related to macroscopic field inhomogeneity effects. High resolution clinical MPRAGE [24] images (1 mm^3^) were also collected for tissue segmentation.

### Image processing

Multi-channel images were combined using a published algorithm [13], and fitted by a model [16] on a voxel-by-voxel basis:

$$S\left(TE\right)=S_{0}\cdot \exp\left[-{R2}_{t}^{*}\cdot TE\right]\cdot F_{BOLD}\left(TE\right)\cdot F\left(TE\right)$$ (1)

where *S*_0_ is the signal magnitude. Functions *F*_BOLD_ and *F* describe signal decay due to BOLD effect [16] and macroscopic field inhomogeneity [21], respectively. This model and data acquisition method allow measurements of tissue-specific R2t* relaxation rate constant free of artifacts related to macroscopic field inhomogeneities and physiological fluctuations.

MPRAGE images were put into “FreeSurfer” (Laboratory for Computational Neuroimaging, Martinos Center for Biomedical Imaging) for brain segmentations, which were registered onto GEPCI maps using “FSL” (FMRIB, Oxford). Median R2t* values for hippocampus, amygdala, parahippocampal cortex, insula, entorhinal cortex, lateral and medial orbitofrontal cortices, caudal and rostral anterior cingulate cortices, isthmus of cingulate, and posterior cingulate were collected. Medians (rather than means) were used to help offset any segmentation errors. Volumes (V) of each limbic region were normalized to skull size using “Sienax” in “FSL” to derive normalized volume (NV) [22]. MPRAGE and FLAIR images were used to obtain lesion load (LL) of MS patients by using “lesion-TOADs” tool [25] in MIPAV [26].

### Clinical neurological tests

The Expanded Disability Status Scale (EDSS), 25 foot timed walk (25FTW), upper extremity function (nine hole peg test - 9HPT), the PASAT, a test of auditory information processing speed and calculation ability, and SDMT, a test of visual processing speed, each validated for MS, were assessed on the day of MRI by examiners blinded to imaging results. Notably, new learning and memory abilities have been previously shown in MS patients to be primarily associated with processing speed [27]. PASAT and SDMT raw scores were converted into z-scores using published control data for age and education.

### Statistics

Analyses were done in the statistical program R. ANCOVA for repeated measures with false discovery rate (FDR) correction, with age and gender as covariates, was used to compare R2t* and NV differences in hippocampus and amygdala between HC and MS patients of different clinical subtypes. R2t* and NV in limbic structures were examined using Pearson correlations with cognitive test scores (PASAT and SDMT), controlled for age, gender, LL and treatment. Spearman correlations, controlled for age, gender, LL and treatment, were used for correlations between R2t* and NV in hippocampus and amygdala, and non-normally distributed clinical results (EDSS, 25FTW and 9HPT). For determining correlations of brain imaging with 9HPT tests, only right-handed (73 of 80 patients) patients were included. 9HPT scores of dominant and non-dominant hands were correlated with MRI measurements in the left and right hemispheres, respectively. FDR with Benjamini-Hochberg procedure [28] was used to correct for multiple testing. After correction, p <0.05 was considered significant.

## Results

### Gender effects on R2t* and volumes in the limbic system

**Fig 1:**
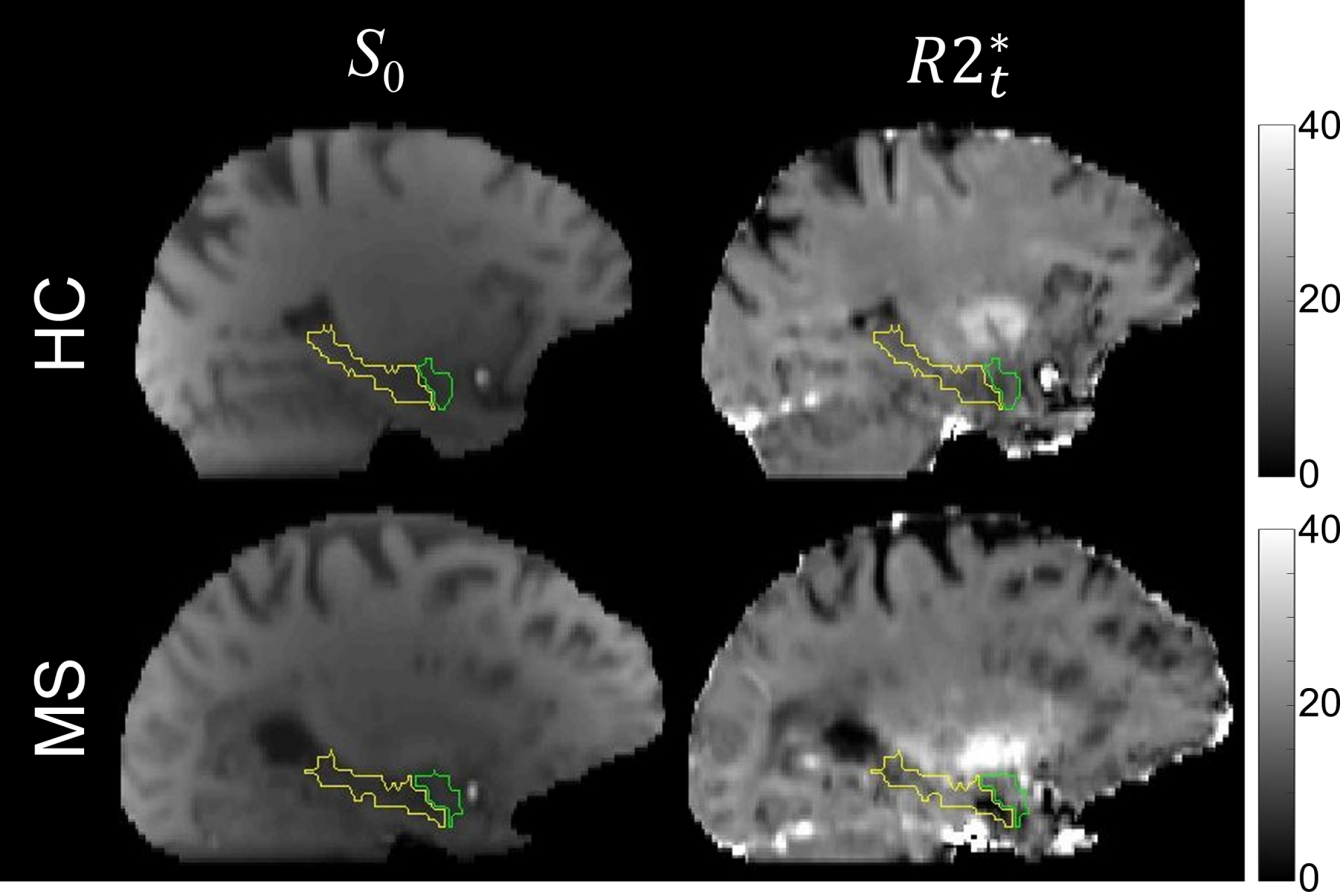
Examples of GEPCI *S*_0_ (T1w) images and R2t* maps selected from a healthy control (HC: 58 yr old, female) and MS patient (MS: 60 yr old, female). Hippocampus and amygdala regions are marked by yellow and green contours, respectively. Despite no dramatic volumetric changes between HC and MS in these two structures, the MS patient showed significant lower R2t* in the anterior hippocampus region.

The limbic system, with focus on the hippocampus and amygdala, was examined using R2t* and regional volumes (Fig 1*)*. Some prior studies reported gender differences in hippocampus size [29,30] and larger amygdala in men [31]. Our data are in agreement with these studies, however these differences do not exist in HC if volumes are normalized to the skull size.

**Fig 2:**
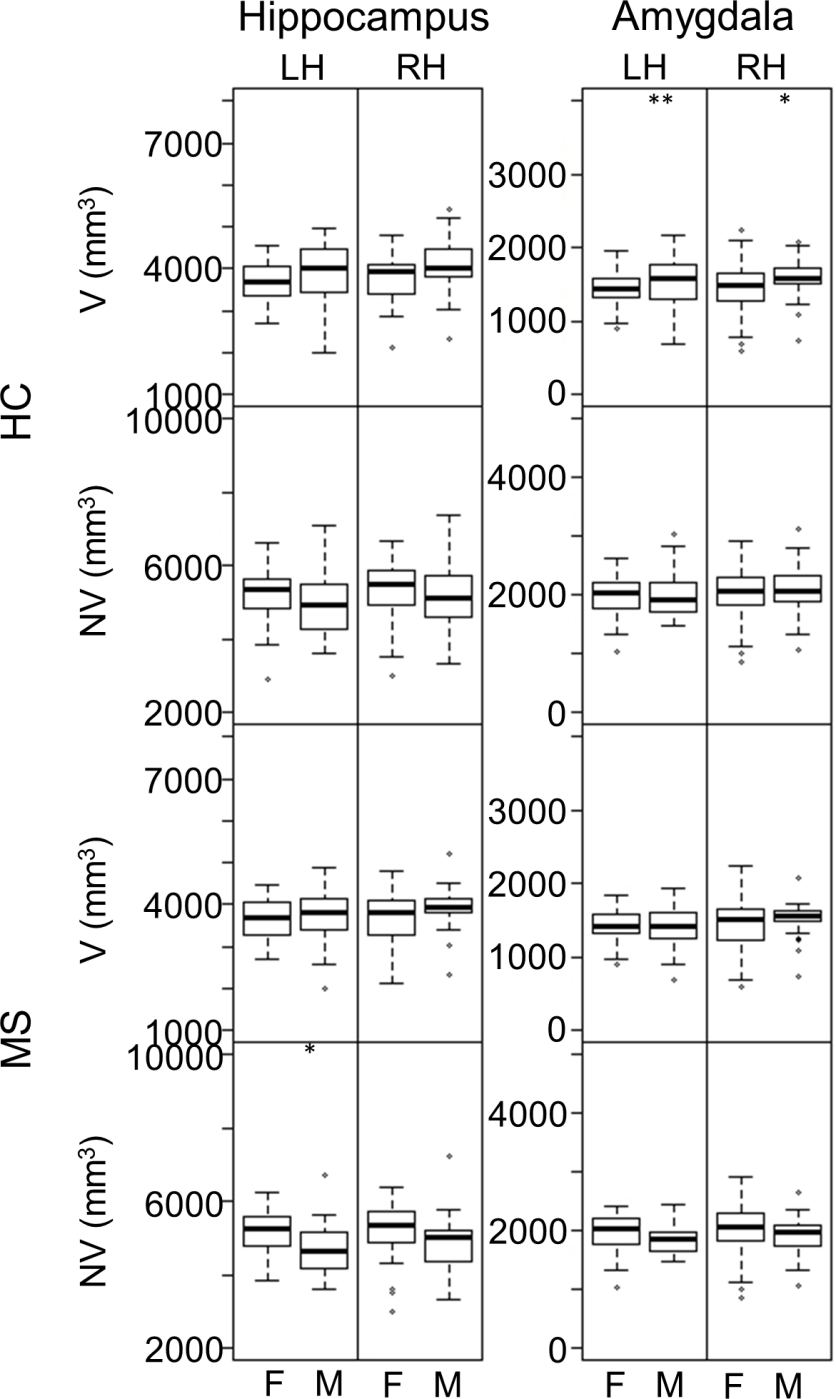
**Gender comparisons for V and NV of hippocampus and amygdala in each hemisphere in HC (A–H) and MS (I–P) groups.** Panels with p-values (with FDR correction) ≤ 0.05 and > 0.01 are marked with “*”. Panels with p-values (with FDR correction) ≤ 0.01 are marked with “**”. F = Female; M = Male; LH = Left Hemisphere; RH = Right Hemisphere.

In HC, we found no significant gender differences in R2t* of hippocampus, amygdala and other limbic cortical regions. In our MS cohort, there were no significant differences in NV between genders except that normalized hippocampal volume was larger in females with MS (Fig 2). Our data do not support a gender difference in normalized size of amygdala, nor do they indicate any gender differences in R2t*.

### Comparisons of R2t* and NV between MS patients and HC

**Fig 3:**
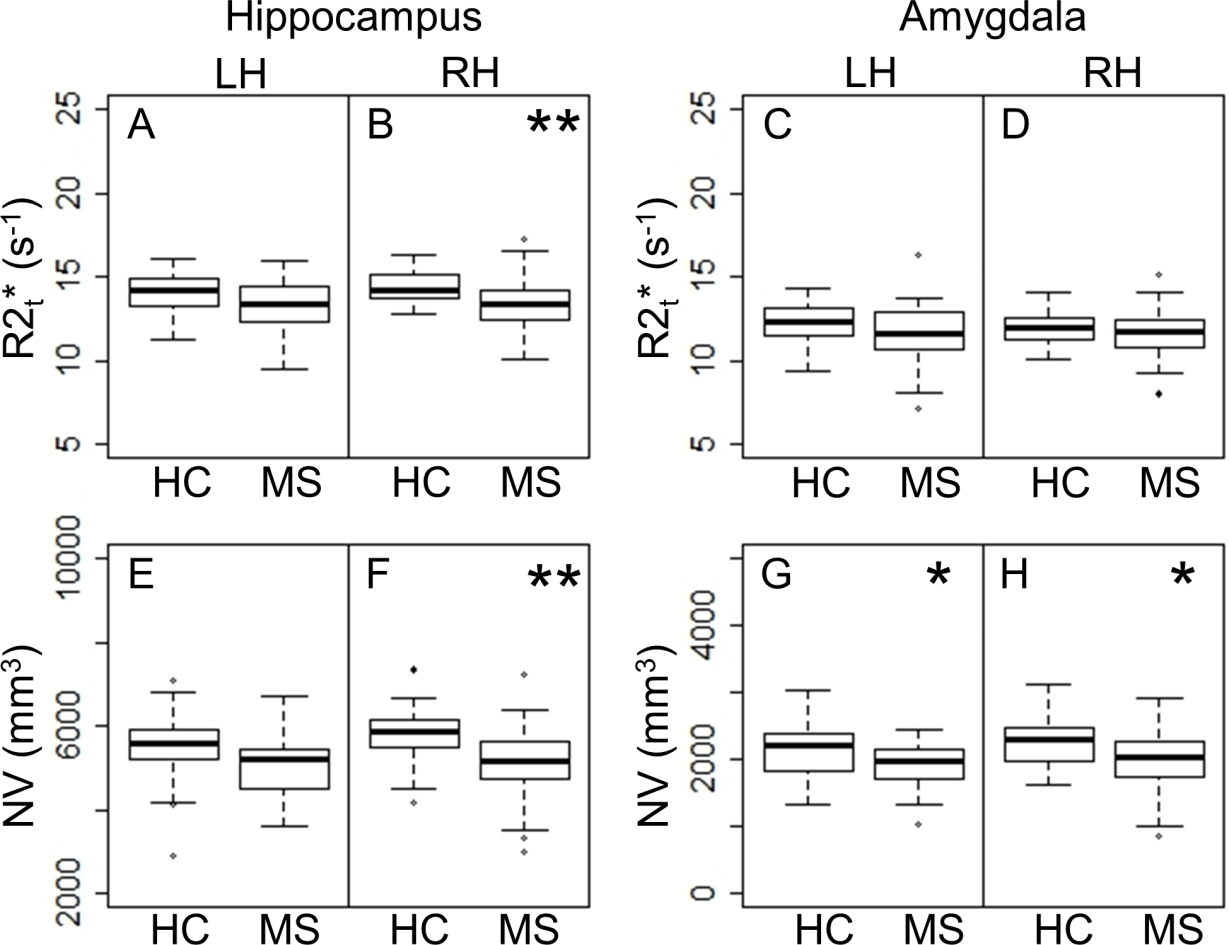
HC versus MS group comparisons of R2t* (A–D) and NV (E–H) of hippocampus and amygdala for each hemisphere. Panels with p-values (with FDR correction) ≤ 0.05 and > 0.01 are marked with “*”. Panels with p-values (with FDR correction) ≤ 0.01 are marked with “**”. LH = Left Hemisphere; RH = Right Hemisphere.

Reduced NV within regions of the limbic system has previously been reported in MS [5–7]. Consistent with those reports, we observed lower NV in MS than HC in both hippocampus and amygdala (Fig 3). MS patients also had lower R2t* in the right hippocampus (Fig 3).

**Fig 4:**
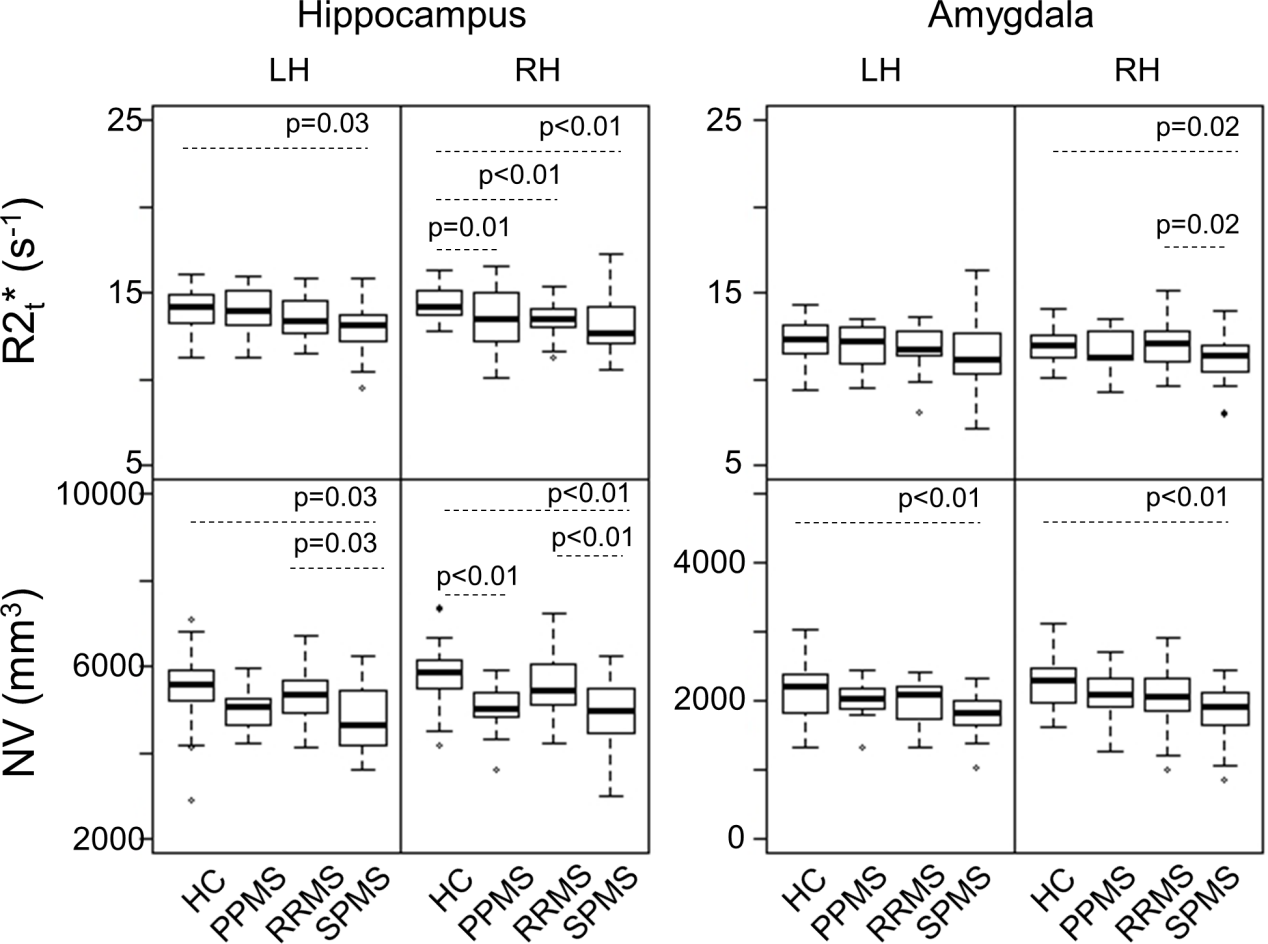
HC versus different MS sub-groups comparisons of R2t* (top) and NV (bottom) of hippocampus and amygdala for each hemisphere. Significant p-values (with FDR correction) are listed in each panel. LH = Left Hemisphere; RH = Right Hemisphere.

MS patients had been categorized as RRMS, SPMS, or PPMS prior to entering the study. We compared R2t* and NV among the clinical subtypes and compared each subtype with HC. All subtypes had reduced R2t* compared to HC in the right hippocampus (Fig 4). R2t* and NV were reduced in SPMS patients in both hippocampus and amygdala compared to HC, except R2t* in left amygdala. PPMS patients had smaller NV in bilateral hippocampus. R2t* of right amygdala and NV of bilateral hippocampus were reduced in SPMS compared to RRMS. (Fig 4).

### Associations of R2t* and NV in hippocampus and amygdala with cognitive test performance

**Fig 5:**
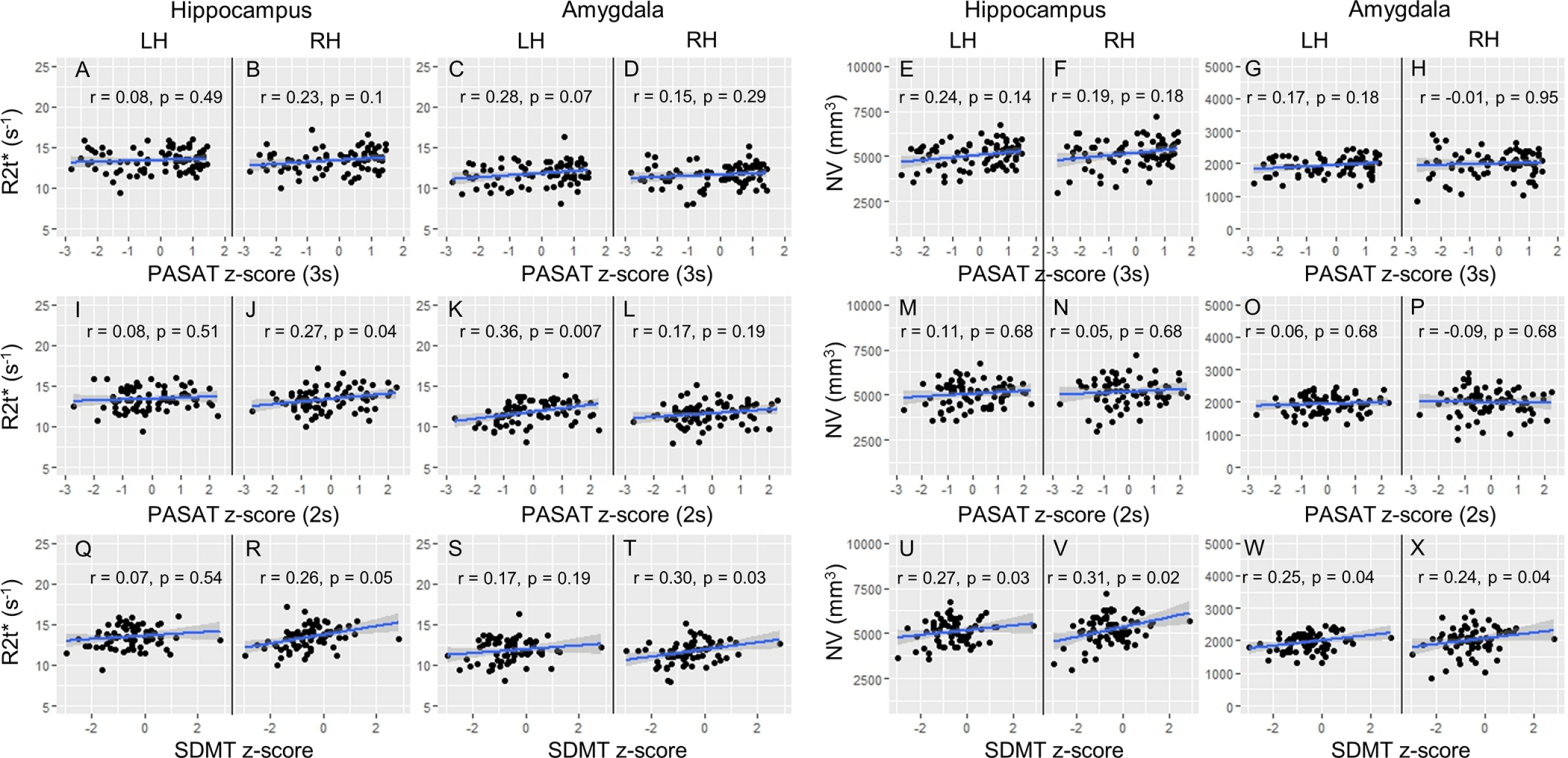
Correlations of R2t* and NV with clinical cognitive scores in hippocampus and amygdala of MS patients. Correlations of R2t* are shown on the left panels. Correlations of NV are shown on the right panels. Correlations with three clinical cognitive tests (3s PASAT, 2s PASAT and SDMT) are presented in 3 rows, respectively. The r-values and p-values (with FDR correction) are provided in each panel. LH = Left Hemisphere; RH = Right Hemisphere.

R2t* and NV of limbic regions were examined for associations with performance on cognitive tests. Reduced R2t* of the right hippocampus and right amygdala each correlated moderately with worse SDMT scores (Fig 5R and 5T). Smaller NV of the hippocampus and amygdala of each hemisphere correlated moderately with worse SDMT performance (Fig 5U–5X). Reduced R2t* in left amygdala correlated moderately with worse cognitive test performance on the 2s PASAT (Fig 5K), however, NV showed no significant correlations with PASAT scores (Fig 5E–5H and 5M–5P).

### Associations of R2t* and NV with tests of physical impairment

**Fig 6:**
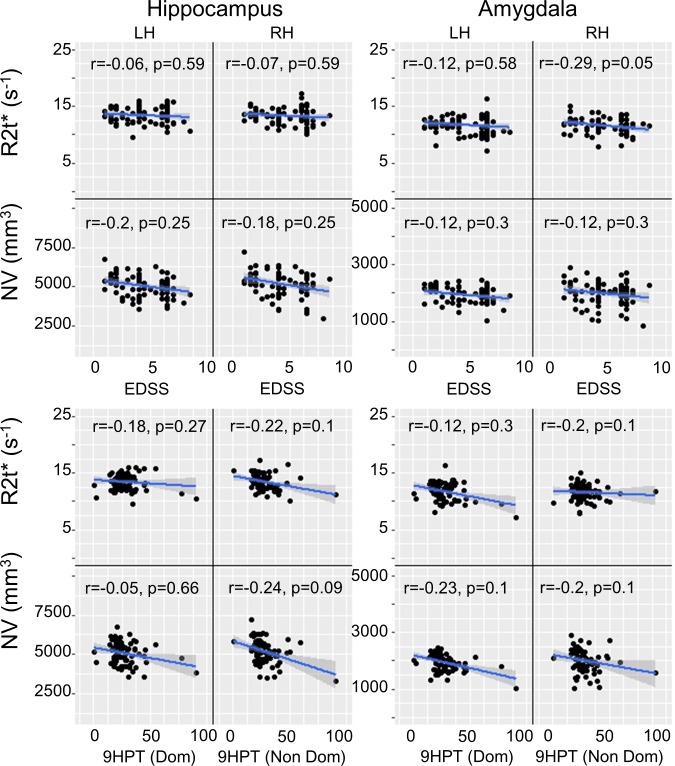
Correlations of R2t* and NV with clinical disability scores (EDSS, 9HPT of dominant and non-dominant hands) in hippocampus and amygdala of MS patients. The correlations (r) and p-values (with FDR correction) are provided in each panel. LH = Left Hemisphere; RH = Right Hemisphere.

Reduced R2t* of right amygdala showed modest correlation with worse EDSS score (r = -0.29, p = 0.05) (Fig 6). Smaller NV of right hippocampus displayed a nonsignificant trend to be correlated with worse non-dominant hand performance on 9HPT (r = - 0.24, p = 0.09).

### Other limbic system structures

We also examined R2t* and NV in other cortical GM regions in the limbic system, including the parahippocampus, insula, and entorhinal, orbitofrontal and cingulate cortices. No significant gender differences in R2t* or NV were found in these structures for HC or MS. Compared to HC, our cohort of MS patients had significantly lower R2t* in the right insula (p<0.01) and left lateral orbitofrontal cortex (p<0.01). In contrast, no differences were found for NV between HC and MS groups in any of these structures. Considering left and right regions as distinct, lower R2t* of 11 limbic cortex regions correlated with worse performance on cognitive tests (bolded, Table 2), whereas no correlations were found for NV. FDR was used to correct for multiple comparisons in all assessments.

**Table 2:** Correlations between clinical cognitive tests and R2t* and NV in limbic cortex.

		R2t*						NV					
		3s PASAT		2s PASAT		SDMT		3s PASAT		2s PASAT		SDMT	
		r	P	r	p	r	p	r	p	r	p	r	p
**Parahippocampal**	LH	0.01	0.91	0.06	0.63	0.13	0.34	0.07	0.77	-0.02	0.98	0.30	0.09
	RH	**0.32**	**0.03**	0.22	0.11	0.24	0.07	0.15	0.77	0.06	0.98	0.33	0.08
**Insula**	LH	**0.31**	**0.03**	0.27	0.05	**0.37**	**0.01**	0.15	0.77	0.12	0.97	0.14	0.31
	RH	0.23	0.08	0.23	0.11	**0.35**	**0.01**	0.07	0.77	0.00	0.98	0.12	0.35
**Entorhinal**	LH	-0.17	0.22	-0.16	0.19	-0.07	0.61	0.11	0.77	0.08	0.98	0.23	0.11
	RH	-0.13	0.34	-0.19	0.13	-0.07	0.61	0.05	0.81	0.02	0.98	0.15	0.31
**Lateral orbitofrontal**	LH	0.21	0.11	0.19	0.14	0.19	0.15	0.10	0.77	0.00	0.98	0.21	0.15
	RH	**0.31**	**0.03**	**0.35**	**0.01**	**0.34**	**0.01**	0.09	0.77	0.04	0.98	0.17	0.24
**Medial orbitofrontal**	LH	0.16	0.22	0.15	0.23	0.14	0.31	0.12	0.77	0.19	0.91	0.12	0.35
	RH	0.09	0.47	0.19	0.13	0.01	0.93	0.02	0.90	0.10	0.98	0.02	0.88
**Caudal anterior cingulate**	LH	**0.36**	**0.01**	**0.42**	**<0.01**	**0.42**	**<0.01**	0.00	0.97	-0.02	0.98	0.19	0.20
	RH	**0.36**	**0.01**	**0.49**	**<0.01**	**0.33**	**0.01**	0.26	0.51	0.15	0.91	0.24	0.11
**Isthmus of cingulate**	LH	0.29	0.03	**0.33**	**0.01**	0.29	0.02	0.02	0.90	0.08	0.98	0.27	0.10
	RH	0.29	0.03	**0.33**	**0.01**	**0.40**	**<0.01**	0.09	0.77	0.12	0.97	0.13	0.32
**Posterior cingulate**	LH	0.26	0.04	0.19	0.13	**0.35**	**0.01**	-0.09	0.77	-0.02	0.98	0.02	0.88
	RH	0.30	0.03	0.27	0.05	**0.32**	**0.01**	0.05	0.81	0.15	0.91	0.24	0.11
**Rostral anterior cingulate**	LH	0.29	0.03	**0.40**	**<0.01**	0.22	0.10	0.12	0.77	0.16	0.91	0.15	0.31
	RH	0.11	0.38	0.19	0.13	-0.11	0.43	0.08	0.77	0.06	0.98	0.25	0.11

Lower R2t* and NV each correlate with worse performance on clinical cognitive tests scores in several regions of the limbic system (significant values are bolded). All p-values are after multiple comparison correction (FDR).

## Discussion

MRI techniques have been successfully used to detect MS pathology for decades. Current standard clinical sequences include T1-weighted (T1W), T2-weighted (T2W), and fluid attenuated inversion recovery (FLAIR). However, these techniques are not quantitative. In this study, we measured quantitative parameters of R2t* and volume of limbic system components in 31 HC and 80 MS patients. Lower R2t* values and smaller normalized volumes were found in the hippocampus, amygdala and several other limbic system regions in MS patients, compared to HC. Both R2t* and volumetric measures correlated with clinical cognitive tests measuring processing speed, working memory, attention and concentration. Notably, correlations of R2t* were stronger and involved more regions, compared to volumetric measures. Thus, compared with volumetrics, R2t* may detect abnormalities earlier or with greater sensitivity. A quantitative tool to sensitively detect changes in the limbic system and other parts of the brain could be useful to guide clinical decisions regarding the effectiveness of a given MS treatment in an individual patient, and might also be useful in trials of potential new therapies.

MRI plays an important role in diagnosis and management of MS. Conventional T1W and T2W imaging techniques allow visualization of many MS lesions in WM but do not correlate well with MS disability. The image contrast in standard “weighted” MR sequences depends not only on MR relaxation time constants of the tissue but also on parameters of the pulse sequences, making it intrinsically non-quantitative; this likely contributes to weak correlations with disability measures.

Tissue specific (i.e. free of artifacts related to magnetic field inhomogeneities and physiological fluctuations) R2* (1/T2*) relaxation rate constant is a quantitative measurement of the transverse relaxation rate of MRI signal in tissue. It can be considered as a quantitative measure reflecting the reciprocal of the degree of hyperintensity of MS lesions on T2W images. Measuring tissue T2 and T1 relaxation times has correlated well with MS pathology seen at autopsy [32]. Such studies suggested that more quantitative tissue-specific relaxation times might provide more accurate and sensitive reflections of pathology than measuring lesion size on “weighted” images.

Alterations of R2* have been shown to reflect MS pathology, including demyelination [33]. We previously reported that R2* is reduced in MS in CNS WM [18] and cortical GM [14], and that R2* measurements in some cortical GM regions correlated with worse cognitive test scores in MS. Because R2* includes contributions due to neuronal activity—BOLD effect [34], we developed methods [16] to separate the tissue-specific part of R2* (which we call R2t*) from the total R2* values. Previously, we demonstrated that changes in R2t* correlated with age-related changes in cellular density in healthy human brain [16,17] and correlated better than R2* with cognitive tests in Alzheimer’s Disease patients [23]. In studies reported in this paper, we compared regional R2t* to NV for ability to detect and measure tissue abnormalities in limbic GM tissues of healthy and MS subjects, and examined relationships of R2t* and NV with physical and cognitive test scores.

R2t* and NV of the hippocampus and amygdala were compared between female and male MS and HC. In HC, no significant gender differences were found for NV or for R2t*. However, in MS patients in our study, males tended to have smaller hippocampi (NV) than females. A previous study [35] has shown that subcortical gray matter atrophy is more significant in male than in female, although that study did not report atrophy in hippocampus. However, hippocampal atrophy has been reported in MS [5–7].

A novel finding in the present study was that MS patients had lower R2t* values in several regions of the limbic system compared to HC, and lower R2t* values significantly correlated with worse performance on cognitive tests relevant to MS. Abnormally low R2t* was seen in right hippocampus, right insula and left lateral orbitofrontal. In our MS cohort, smaller NV in MS than HC was only noted in the hippocampus and amygdala, and no correlations with cognitive scores were found for volumetric measures. Together, these findings suggest that MS damage is widespread within the limbic system, and that R2t* measurements are more sensitive to tissue damage in these structures than is atrophy. Importantly, our results might also indicate that R2t* can detect changes earlier than volume changes, but longitudinal studies will be needed to confirm this. Since volumetric measurements are the current standard in the field to measure tissue damage related to neurodegeneration in MS, this study will provide a quantitative tool with considerable potential to detect abnormalities in the limbic system with more sensitivity and/or earlier.

The amygdala and hippocampus are located in the medial temporal lobe, a region which is involved in recognition of sounds [4]. We observed that reductions of R2t* in both hippocampus and amygdala in MS patients correlated with worse results on the PASAT, a test of auditory information processing speed, calculation ability, and working memory. Correlations with 2s PASAT (r = 0.36, p = 0.007) were stronger than with 3s PASAT (r = 0.28, p = 0.07) in left amygdala. This might reflect the greater difficulty of the 2s PASAT, making the latter more sensitive to mild abnormalities. The amygdala is well known to function in emotional learning and memory [36]. The 2s PASAT is considered more stressful than the 3s PASAT, perhaps evoking an emotional response in the test-taker that is sensitive to amygdala abnormalities. In contrast, NV of the hippocampus and amygdala only correlated with SDMT, but not with 3s or 2s PASAT scores. Interestingly, regional correlations of NV and R2t* with cognitive test results did not always overlap. These disparate results indicate that R2t* can contribute information on tissue integrity that volume does not. We found weak or no correlations between R2t* in limbic system and motor functions tested by 9HPT and 25TW, respectively. This was not unexpected. We suspect any weak correlations with 9HPT are indirect–that is, more a result of the global level of CNS pathology in a given patient than to a direct structure-function correlation.

There are several limitations in the current study. The number of participants was relatively small, which might have limited the ability to detect correlations. Extensive tests of memory, learning and emotion were not included in our study design. We did find that R2t* and volumetric measures showed significant correlations with tests that are sensitive to common MS cognitive deficits (PASAT and SDMT), including processing speed and working memory. Future studies will include more thorough testing of the primary hippocampus and amygdala functions of memory and learning.

## Conclusions

This study reports several novel findings, as well as expanding on existing literature. Limbic system abnormalities have received relatively little prior attention in MS. We found reduced R2t*, a measure that reflects tissue integrity, in several cortical limbic system regions that were not abnormal using volumetrics. R2t* was also more sensitive to changes in cognitive test performance than NV. High resolution MR sequences to measure R2t* require less than 15 minutes, and can be readily added to scanning protocols on clinical scanners. This and prior studies indicate that R2t* can be used as a quantitative tissue biomarker, providing information that is distinct from and complementary to volume.
